# Current Status of Social Problems during Pregnancy at a Perinatal Center in Japan

**DOI:** 10.31662/jmaj.2020-0056

**Published:** 2020-10-02

**Authors:** Shunji Suzuki, Masako Eto

**Affiliations:** 1 Department of Obstetrics and Gynecology, Japanese Red Cross Katsushika Maternity Hospital, Tokyo, Japan

**Keywords:** social problems, pregnancy, perinatal center, Japan

## Abstract

**Introduction:**

To support pregnant women with serious social problems, we retrospectively examined the current status of social problems during pregnancy in Japan.

**Methods:**

We examined the frequency, associated factors, and outcomes of the pregnant women with social problems at our institute for each year from 2016 to 2019.

**Results:**

The frequency of pregnant women with social problems significantly increased year by year (*p* < 0.01). The breakdown of high-risk factors associated with the social problems has remained almost unchanged; however, the frequency of unmarried pregnant women, unplanned pregnancy, foreigners who cannot speak either Japanese or English, and pregnant women who are somehow anxious significantly increased (*p* < 0.05).

**Conclusions:**

We will endeavor to solve the various social problems of pregnant women through multidisciplinary collaboration.

## Introduction

Recently, the increased number of child abuse has become one of the serious problems in Japan^(1)^. Although Japan had not been believed to be a poor country, the number of isolated mothers has been increased dramatically due to the increased economic wealth gap, increased nuclear families and the collapse of local communities in Japan^(1), (2)^. Although child abuse can occur anytime and anywhere, “specified expectant mothers” have been defined as pregnant women at high risk of abuse and in need of extra support after birth because of unstable income, mental disorders, etc., in 2010 by the Japanese Ministry of Health, Labour and Welfare^(3)^. Our institute is one of the main Japanese perinatal centers (about 1,700 deliveries per year) located in a downtown area of Tokyo. At our institute, the pregnancies and deliveries of at least 200 specified expectant mothers per year were managed.

In the area of Tokyo where our facility is located, the following pregnant women are recognized as “specific expectant mother”^(3), (4)^: (1) pregnant women who had not been examined until mid-pregnancy and those who have not prepared for childbirth, (2) teenage pregnant women, (3) unmarried pregnant women, (4) pregnant women who had problems with their previous children, (5) pregnant women with mental disorders, (6) women with unwanted pregnancy and/or conflict with pregnancy, (7) pregnant women with poverty and/or unemployed partners, (8) pregnant women of foreign nationality, (9) pregnant women receiving intimate partner violence (IPV), (10) pregnant women who cannot expect childcare support around them, (11) pregnant women who are thought to be anxious such as those who are aggressive or impulsive, (12) pregnant women with alcohol or drug dependence, (13) pregnant women who move frequently from one place to another, and (14) women with children with problems such as premature babies and disabled children.

We have supported their social problems through multidisciplinary collaboration composed of midwives, doctors, clinical psychologists, medical social workers, and regional administrative staff during pregnancy and postpartum ^(5), (6), (7)^. For example, clinical psychologists evaluate the mental status and give advice on how to relate to psychiatrists and/or regional staff. The medical social workers provide support and information on social resources and serve as contact points for regional organizations. The medical treasurers explain financial counseling and procedures. The information about the pregnant women is shared, opinions are exchanged from each specialized area, and support methods are decided. If necessary, we all meet and discuss with psychiatrists, pediatricians, and regional administrative staff directly.

To further strengthen our support for specific expectant mothers, we retrospectively examined the current status of their social problems and the effect of our support.

## Materials and Methods

The study protocol was approved by the Ethics Committee of the Japanese Red Cross Katsushika Maternity Hospital (K2019-26). Informed consent concerning retrospective analyses was obtained from all subjects.

**Figure 1.**
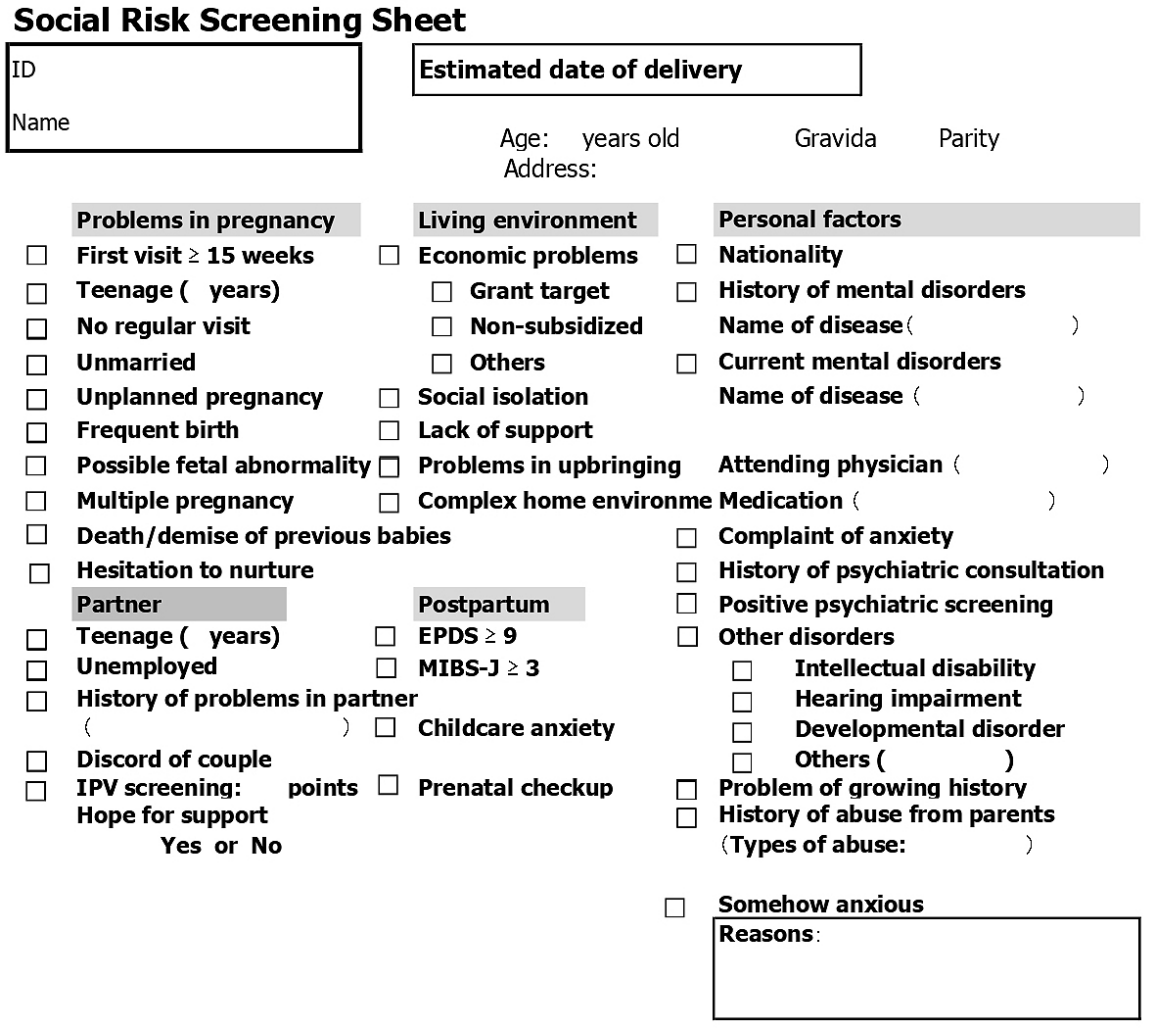
Social Risk Screening Sheet used at Japanese Red Cross Katsushika Maternity Hospital *Japanese version is used. EPDS, the Edinburgh Postnatal Depression Scale; MIBS-J, the Japanese version of Mother-to-Infant Bonding Scale; IPV, intimate partner violence.

In our institute, midwives conduct three health consultations during pregnancy to support the healthy lives of pregnant women^(7), (8)^. These consultations were at approximately 8-11, 20-23, and 34-36 weeks' gestation. At the consultations, the social and economic information has also been obtained from all pregnant women using our original questionnaire (in Japanese), as shown in Figure 1.

During pregnancy, mental status has been checked by the following three screening tools^(8), (9)^: (1) the modified Violence Against Women Screen^(9), (10)^, a Japanese screening instrument for IPV to identify pregnant women who have experienced abuse; (2) the tale of Whooley questions^(11), (12)^, a screening instrument for depression in the general adult population including pregnant and postpartum women; and (3) the 2-item Generalized Anxiety Disorder Scale to screen for generalized anxiety disorder^(12), (13)^. In this study, we considered women with positive mental screening as those without risk factors of mental disorders^(7)^.

In Japan, the hospitalization assistance policy (HAP) system, based on the Child Welfare Act, assists pregnant women who, for financial reasons, cannot give birth at medical institutions^(14)^. The HAP system allows these women to deliver at specified (midwifery) institutions. The main objectives of the HAP system are to help pregnant women who (1) receive the livelihood protection because they are unable to maintain minimum living standards because of poverty, (2) live in households exempt from the residence tax, and (3) live in households in which the income tax is less than ¥8,400 (equivalent to approximately 100 US dollars) per year.

In this study, we examined the frequency, associated factors, and outcomes of the specific expectant mothers (= pregnant women with at least one social risk factor) managed at our institute for each year from 2016 to 2019. Data are expressed as numbers and percentages. One-way analyses of variance were used. Differences with *p* < 0.05 were considered significant.

## Results

Table 1 shows the frequency of specific expectant mother managed at our institute each year. The frequency of the specific expectant mother significantly increased year by year (*p* < 0.01).

**Table 1.** Frequency of the Specific Expectant Mother Managed at Japanese Red Cross Katsushika Maternity Hospital from 2016 to 2019.

Year	Total	Specific expectant mother
2016	1,885	208 (11.0)
2017	1,863	279 (15.0)
2018	1,745	372 (21.3)
2019	1,650	435 (26.4)

Data are presented as number (percentage).

**Figure 2.**
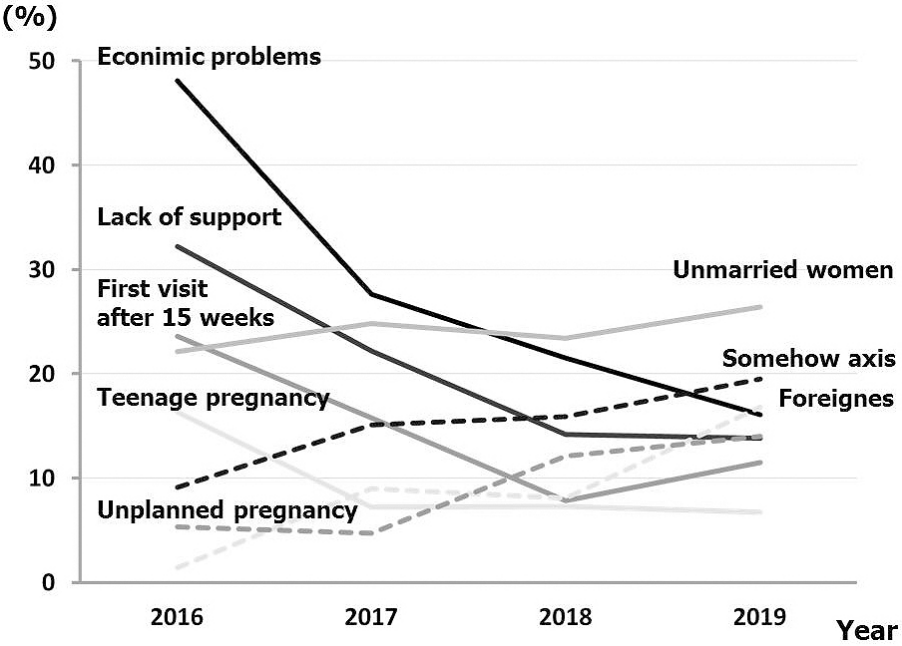
Changes in the frequency of main social factors associated with the specific expectant mother at Japanese Red Cross Katsushika Maternity Hospital from 2016 to 2019 (duplicate).

Table 2 shows the number (frequency) of main social factors associated with the specific expectant mother in each year (duplicate). The most common social problems faced by pregnant women in 2016 were (1) economic problems, (2) lack of support, (3) first prenatal visit after 15 weeks of gestation, (4) unmarried pregnancy, and (5) teenage pregnancy. On the other hand, in 2019, they were (1) unmarried women, (2) somehow anxious, (3) having an unplanned pregnancy, (4) having an economic problem, and (5) foreigners who cannot speak either Japanese or English. These changes are shown in Figure 2.

**Table 2.** Main Social Factors Associated with the Specific Expectant Mother from 2016 to 2019 (Duplicate).

Year	2016	2017	2018	2019
Total	1,885	1,863	1,745	1,650
Specific expectant mother	208	279	372	435
Social factor
First visit at ≥ 15 weeks	49 (23.6)	44 (15.8)	29 (7.8)	50 (11.5)
Teenage	34 (16.3)	20 (7.2)	27 (7.3)	29 (6.7)
No regular visit	17 (8.2)	20 (7.2)	15 (4.0)	6 (1.4)
Unmarried	46 (22.1)	69 (24.8)	87 (23.4)	115 (26.4)
Unplanned pregnancy	3 (1.4)	25 (9.0)	30 (8.1)	73 (16.8)
Partner
Teenage	13 (6.3)	6 (2.2)	9 (2.4)	12 (2.8)
Unemployed	20 (9.6)	13 (4.7)	9 (2.4)	18 (4.1)
Initiate partner violence	30 (14.4)	50 (17.9)	66 (17.7)	51 (11.7)
Economic problems
Grant target	67 (32.2)	55 (19.7)	55 (14.8)	44 (10.1)
Non-subsidized	33 (15.9)	22 (7.9)	25 (6.7)	26 (6.0)
Lack of support	67 (32.2)	62 (22.2)	53 (14.2)	60 (13.8)
Mental disorders	30 (14.4)	41 (14.7)	37 (9.9)	38 (8.7)
Other disorders	16 (7.7)	5 (1.8)	6 (1.6)	9 (2.1)
Positive of mental screening*	13 (6.3)	17 (6.1)	21 (5.8)	28 (6.4)
Foreigners who cannot talk	11 (5.3)	13 (4.7)	45 (12.1)	61 (14.0)
Somehow anxious	19 (9.1)	42 (15.1)	59 (15.9)	85 (19.5)

Data are presented as number (percentage). * Positive of mental screening without risk factors of mental disorders only.

The number of unmarried pregnant women, unplanned pregnancies, foreigners who cannot speak either Japanese or English, and pregnant women with somehow anxious increased significantly (*p* < 0.05) year by year. The main factors leading to anxiety were as follows: (1) behavioral concerns such as several troubles and complaints at the reception, a strong feeling of obstinacy, and poor facial expression without looking in the eye; (2) feeling discomfort because of their partner's behavior; (3) mark of self-harm; (4) poor relationship with her mother; (5) low self-affirmation; and (6) dislike of medical intervention.

Table 3 shows the occupations supporting the social factors associated with specific expectant mothers (duplicate) and the number of mothers unable to raise children for each year. In our institute, the percentage of required occupations varied each year. The number of mothers who have difficulty raising children has decreased over the last 2 years.

**Table 3.** Support Occupations and Mothers Unable to Raise Children from 2016 to 2019.

Year	2016	2017	2018	2019
Number of specific expectant mothers	208	279	372	435
Medical social workers	123 (59.1)	171 (61.3)	157 (42.2)	125 (28.7)
Clinical psychologists	51 (24.5)	92 (33.0)	94 (25.3)	78 (17.9)
Regional administrative staffs	61 (29.3)	87 (31.2)	123 (33.1)	112 (25.7)
Mother unable to raise	7 (3.4)	11 (3.9)	4 (1.1)	2 (0.5)

Data are presented as number (percentage).

## Discussion

Based on the current results, the number/ratio of specific expectant mothers has been increasing year by year. We cannot deny the impact of changes in our attitude toward more active support; however, the environment surrounding Japanese pregnant women may be deteriorating. Japanese people may not be poor on average, but the gap between the wealthy and poor may be widening^(1), (2)^. The current results suggest that pregnant women in Japan may have more severe social and/or economic distress and social isolation than expected.

Although the breakdown of high-risk factors associated with the difficulty of raising children have remained almost unchanged, the increased number of some social problems was observed. However, if once the factors have been identified, they may not be difficult to solve. These might have resulted in the decreased number of women with the difficulty raising children. The proactive but efficient support may also have led to a reduction in the total number of healthcare providers. Some support examples can be provided.

First, the number of unmarried pregnant women and unplanned pregnancies was increased. These are problems of pregnant women before they visit the obstetric institutes. According to a previous study in Tokyo, more than half of the suicides among pregnant women occurred at 2 months of gestation, and they were presumed to be associated with the environment of the women and unplanned pregnancy^(15)^. Therefore, these problems require active intervention and support. During the early stage of gestation, it is necessary to understand the life environment and acceptance status for pregnancy and provide support to fill in the deficits necessary for their life. In some cases, after the first perinatal visit to confirm the pregnancy, they may not be able to continue the pregnancy, cannot take the prenatal visits due to financial problems, and/or feel anxious about pregnancy and parenting. They sometimes cannot keep up with the visit interval. In our institute, we pick them up and send a message to them by e-mail to visit our institute because it is sometimes difficult to approach them on the phone or through direct visit^(6), (7)^.

Second, the number of foreigners who cannot speak either Japanese or English and cannot communicate sufficiently increased. Because of the differences in lifestyle habits such as diet and religion, foreigners seem to be more likely to have mental problems especially during pregnancy and postpartum in Japan. The number of foreigners living in Japan is increasing year by year; however, it is not easy to raise children isolated from the living area due to language barriers. With the increasing number of foreign residents becoming settled, it is necessary to support language and culture in all fields related to life such as work, education, and communication with the community. Even if it is difficult to have a smooth conversation with foreign women, it will be important to become relative and listen to each other.

“Somehow anxious” is an abstract expression; however, the impression of the medical and non-medical staff that pregnant women are somewhat anxious may be very important. For example, the receptionists sometimes tell us about the strange behavior of pregnant women and/or their families in the waiting room. As a trigger for considering the support, reports like “a polite pregnant woman in the examination room sometimes scolds her child violently” will be helpful. The awareness of the importance of the reporting in the staff may have contributed to the recent increase in this factor.

We know that this study has several serious limitations besides a small size of subjects. We have known that it is not possible to consider the current observation as trends for whole pregnant women living in Japan because the social factors have been greatly affected by the region and environment. In addition, we understand that their problems cannot be achieved by the efforts of medical institutions only.

In any case, we will endeavor to solve the social problems of various specific pregnant women through multidisciplinary collaboration.

## Article Information

### Conflicts of Interest

None

### Author Contributions

Shunji Suzuki: project development, data management, data analysis, and manuscript writing/editing

Masako Eto: project development, data collection, data analysis, and manuscript writing/editing

### Approval by Institutional Review Board (IRB)

The study protocol was approved by the Ethics Committee of the Japanese Red Cross Katsushika Maternity Hospital (K2019-26).

### Informed Consent

Patients' informed consent for publication of this report was obtained.
